# Management of severe hypertension due to lenvatinib in patients with advanced thymic carcinoma: A case report

**DOI:** 10.1097/MD.0000000000028476

**Published:** 2022-01-07

**Authors:** Kinnosuke Matsumoto, Takayuki Shiroyama, Kotaro Miyake, Yuji Yamamoto, Tomoki Kuge, Midori Yoneda, Makoto Yamamoto, Yujiro Naito, Yasuhiko Suga, Kiyoharu Fukushima, Shohei Koyama, Kota Iwahori, Haruhiko Hirata, Izumi Nagatomo, Yoshito Takeda, Atsushi Kumanogoh

**Affiliations:** a Department of Respiratory Medicine and Clinical Immunology, Graduate School of Medicine, Osaka University, Yamadaoka, Suita City, Osaka, Japan; b Department of Immunopathology, WPI, Immunology Frontier Research Center (iFReC), Osaka University, Yamadaoka, Suita City, Osaka, Japan; c Integrated Frontier Research for Medical Science Division, Institute for Open and Transdisciplinary Research Initiatives (OTRI), Osaka University, Yamadaoka, Suita City, Osaka, Japan; d Center for Infectious Disease for Education and Research (CiDER), Osaka University, Yamadaoka, Suita City, Osaka, Japan.

**Keywords:** dose reduction, drug interruption, hypertension, lenvatinib, thymic carcinoma

## Abstract

**Rationale:**

Thymic carcinoma (TC) is a malignant mediastinal tumor, and there are no established treatments for pre-treated patients with advanced TC. Recently, lenvatinib was approved for such patients in Japan, ahead of other countries. Higher dose lenvatinib may be more efficacious than conventional treatments, although many patients experience grade 3 hypertension. Therefore, lenvatinib dose reduction remains controversial in terms of efficacy and tolerability.

**Patient concerns:**

Case 1 involves a 72-year-old woman who underwent complete resection of TC and was taking cilnidipine and azilsartan for hypertension. Six years later, multiple lung metastases were observed, and lenvatinib was started. Case 2 involves a 60-year-old man with TC, and was taking amlodipine for hypertension. A chest computed tomography showed progression in primary and metastatic lesions, and the patient started lenvatinib.

**Diagnoses:**

In both patients, grade 3 hypertension was observed after the administration of lenvatinib.

**Interventions:**

In Case 1, lenvatinib dose was reduced 3 times because lenvatinib was not interrupted despite grade 3 hypertension. In contrast, in Case 2, lenvatinib was interrupted when grade 3 hypertension occurred and was resumed after a decrease in blood pressure to baseline.

**Outcomes:**

In Case 2, higher tumor regression may have been achieved because of the maintenance of a high dose of lenvatinib compared with that in Case 1.

**Lessons:**

Lenvatinib is a promising agent for advanced TC; however, hypertension should be addressed cautiously, especially at the outset of administration. Lenvatinib may have to be appropriately interrupted and resumed as soon as the blood pressure is controlled to maximize efficacy and minimize toxicity.

## 1 Introduction

Thymic carcinoma (TC) is a mediastinal malignant tumor that accounts for 12% to 14% of all thymic epithelial tumors.^[1]^ Although surgery is the first-line treatment, most patients are in an advanced stage when diagnosed, which results in a poor prognosis (5-year survival rate of 24.2%).^[2]^ Therefore, cytotoxic chemotherapies are widely used to treat unresectable or metastatic TCs. In terms of efficacy and safety, carboplatin plus paclitaxel is the most recommended first-line treatment for advanced TC.^[3]^ Second-line or higher systemic treatments include sunitinib, pemetrexed, everolimus, paclitaxel, octreotide, 5-fluorouracil, gemcitabine, and etoposide; however, these regimens are not sufficiently efficacious for TC treatment.^[4]^

In March 2021, before other countries, lenvatinib was first approved for pre-treated patients with advanced TC based on the results of a phase 2 trial (REMORA) in Japan.^[5]^ Although lenvatinib showed promising benefits for advanced TC, severe hypertension was reported at a high frequency. Several clinical trials have reported that lenvatinib causes hypertension; the detailed management approaches for lenvatinib doses remain unclear. Here, we present 2 advanced TC patients with hypertension to investigate the optimal dose adjustment of lenvatinib for severe hypertension.

## 2 Case presentation

### 2.1 Case 1

**Figure 1:**
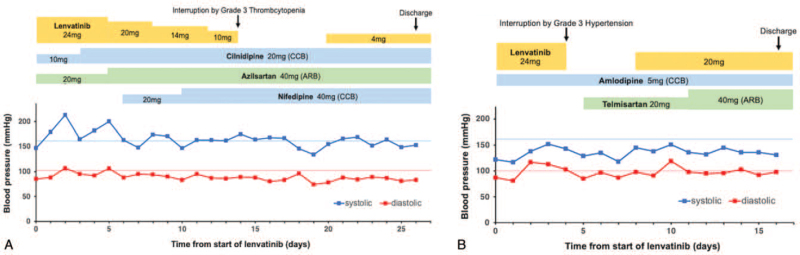
Clinical course of 2 patients with advanced TC who received lenvatinib therapy. (A) Case 1: without lenvatinib interruption, continuous grade 3 hypertension occurred several times, despite dose reduction and aggressive use of antihypertensive agents. (B) Case 2: grade 3 hypertension occurred 3 times; lenvatinib was maintained at a high dose with appropriate drug interruption and aggressive use of antihypertensive agents. ARB = angiotensin receptor blocker, CCB = calcium channel blocker, TC = thymic carcinoma.

**Figure 2:**
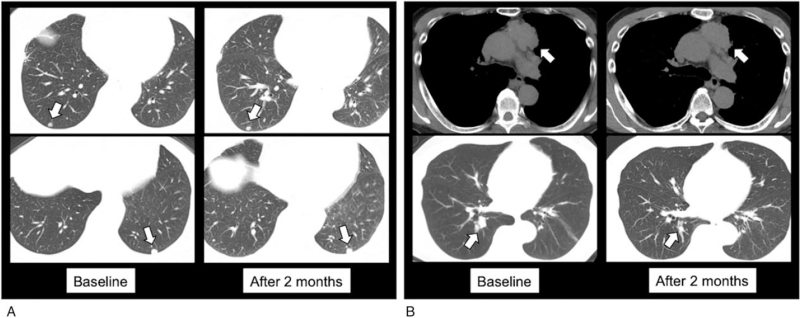
Chest CT scan showing the serial changes at baseline and 2 months after lenvatinib initiation. (A) Chest CT image of Case 1 reveals no remarkable changes in the size of the target lesions (white arrow). (B) Chest CT image of Case 2 presents the decrease in the size of the target lesions (from 80.9–67.1 mm) (white arrow). CT = computed tomography.

A 72-year-old woman, who underwent complete resection of TC 6 years ago, has had controlled hypertension (systolic blood pressure ≤140 mm Hg and diastolic blood pressure ≤100 mm Hg); she had been taking cilnidipine (10 mg/day) and azilsartan (20 mg/day). Multiple lung metastases were newly observed in chest computed tomography (CT), and therefore, lenvatinib (24 mg/day) was initiated. From the next day, the patient experienced continuous grade 3 hypertension (systolic blood pressure ≥160 mm Hg or diastolic blood pressure ≥100 mm Hg), and 3 antihypertensive agents were required to control hypertension (Fig. 1A). Despite lenvatinib dose reduction without interruption and the aggressive use of antihypertensive agents, grade 3 hypertension had recurred several times. Lenvatinib was decreased to 10 mg/day (3 steps of dose reduction) within 2 weeks of treatment initiation, and finally, interrupted by grade 3 thrombocytopenia, which was completely ameliorated with interruption alone. Lenvatinib was resumed at 4 mg/day, as her blood pressure dropped with the 3 antihypertensive agents. Two months after lenvatinib initiation, a chest CT revealed no remarkable changes in the existing lung metastases (Fig. 2A). After lenvatinib resumption, grade 3 and higher hypertension was not observed for 4 months, but the patient has been using the 3 antihypertensive drugs.

### 2.2 Case 2

A 60-year-old man, who was diagnosed with TC by thoracoscopic biopsy, had been taking amlodipine (5 mg/day), and his hypertension was under control (systolic blood pressure ≤140 mm Hg and diastolic blood pressure ≤100 mm Hg). A chest CT showed the progression of primary lesion and increase in pulmonary and pleural metastases. Thus, lenvatinib (24 mg/day) was commenced as a second-line treatment. He experienced grade 3 hypertension (diastolic blood pressure ≥100 mm Hg) 2 days later, and lenvatinib was interrupted once (Fig. 1B). As his blood pressure dropped to the baseline level, lenvatinib was resumed at 20 mg/day. Lenvatinib at 20 mg/day was then maintained, along with 2 antihypertensive agents. Two months after the start of lenvatinib, a chest CT revealed a decrease in size of the target lesions (from 80.9–67.1 mm) (Fig. 2B). Grade 3 and higher hypertension was not observed with lenvatinib (20 mg/day) and 2 antihypertensive agents 4 months after lenvatinib resumption.

## 3 Discussion

Herein, we have presented the clinical course of 2 patients with advanced TC who received lenvatinib. A *post hoc* analysis of data of the phase 3 trial (SELECT) for thyroid cancer revealed that shorter interruptions of lenvatinib provided more favorable survival benefits.^[6]^ Therefore, we compared patients who did not and did experience interruption in lenvatinib. To the best of our knowledge, this is the first report on drug interruption in patients with advanced TC, especially with pre-existing hypertension.

In the REMORA trial, lenvatinib demonstrated an objective response rate of 38% and a disease control rate of 95% in patients with previously treated advanced TC.^[4]^ Although lenvatinib showed more promising efficacy than existing drugs, all patients experienced at least 1 level of dose reduction because of adverse events (AEs), and over 80% of the patients experienced 3 or more dose reductions.

Lenvatinib dose reduction remains controversial in terms of efficacy and tolerability. In another exploratory analysis of SELECT, a higher dose of lenvatinib contributed to greater tumor size reduction in the first 8 weeks.^[7]^ In the REMORA trial, most patients also showed tumor regression when taking 20 to 24 mg/day at first 8 weeks after administration.^[4]^ These studies indicate that maximal benefits may be obtained by continuing the administration of lenvatinib at a dose as high as possible.

However, in the REMORA trial, the most common grade ≥3 AE was hypertension (64%), leading to dose reduction (24%). These figures cannot be overlooked and are similar to the results of the SELECT trial; the most frequent grade ≥3 AE was hypertension (41.8%), leading to dose reduction (19.9%).^[8]^ Thus, hypertension is one of the most notable AEs associated with dose reduction. Moreover, in the REMORA trial, the median time to the first occurrence of hypertension was 8 days, which was earlier than the occurrence of any other toxicity, and 80% of the patients experienced hypertension within 8 weeks after lenvatinib administration.^[4]^ These findings suggest that appropriate management of hypertensive toxicity is also essential for early treatment.

In Case 1, lenvatinib was continued without interruption to increase the dose intensity, which led to 3 dose reductions, resulting in no remarkable changes in tumor size. In Case 2, lenvatinib was appropriately interrupted and resumed after the blood pressure dropped; overall, a high dose was maintained and tumor size decreased. Thus, lenvatinib may have to be appropriately interrupted and resumed as soon as blood pressure is controlled to maximize efficacy and minimize toxicity.

In conclusion, this report highlights the importance of appropriate interruption of lenvatinib in hypertension patients with advanced TC. Lenvatinib is a promising agent for suppressing tumor progression, whereas hypertension should be addressed appropriately with drug interruption and antihypertensive agents, especially during the first 8 weeks of treatment initiation. Further studies are warranted to confirm the appropriate management of lenvatinib dosage in clinical settings.

## Author contributions

**Conceptualization:** Kinnosuke Matsumoto, Takayuki Shiroyama.

**Data curation:** Kinnosuke Matsumoto, Takayuki Shiroyama.

**Investigation:** Kinnosuke Matsumoto, Takayuki Shiroyama.

**Project administration:** Takayuki Shiroyama, Atsushi Kumanogoh.

**Resources:** Kinnosuke Matsumoto, Takayuki Shiroyama.

**Supervision:** Takayuki Shiroyama, Atsushi Kumanogoh.

**Writing – original draft:** Kinnosuke Matsumoto.

**Writing – review & editing:** Kinnosuke Matsumoto, Takayuki Shiroyama, Kotaro Miyake, Yuji Yamamoto, Tomoki Kuge, Midori Yoneda, Makoto Yamamoto, Yujiro Naito, Yasuhiko Suga, Kiyoharu Fukushima, Shohei Koyama, Kota Iwahori, Haruhiko Hirata, Izumi Nagatomo, Yoshito Takeda, Atsushi Kumanogoh.

## References

[1] Nakajima J Okumura M Yano M. Myasthenia gravis with thymic epithelial tumour: a retrospective analysis of a Japanese database. Eur J Cardiothorac Surg 2016;49:1510–5. 26537756 10.1093/ejcts/ezv380

[2] Kondo K Monden Y. Therapy for thymic epithelial tumors: a clinical study of 1,320 patients from Japan. Ann Thorac Surg 2003;76:878–84. discussion 884. 12963221 10.1016/s0003-4975(03)00555-1

[3] National Comprehensive Cancer Network Clinical Practice Guidelines in Oncology. Thymomas and Thymic Carcinomas, version 1. 2021. Available at: https://www.nccn.org/professionals/physician_gls/pdf/thymic.pdf. Accessed September 14, 2021.

[4] Merveilleux du Vignaux C Dansin E Mhanna L. Systemic therapy in advanced thymic epithelial tumors: insights from the RYTHMIC prospective cohort. J Thorac Oncol 2018;13:1762–70. 30138763 10.1016/j.jtho.2018.08.005

[5] Sato J Satouchi M Itoh S. Lenvatinib in patients with advanced or metastatic thymic carcinoma (remora): a multicentre, phase 2 trial. Lancet Oncol 2020;21:843–50. 32502444 10.1016/S1470-2045(20)30162-5

[6] Tahara M Brose MS Wirth LJ. Impact of duration of dose interruption on the efficacy of lenvatinib in phase 3 study in patients with radioiodine-refractory differentiated thyroid cancer. Eur J Cancer 2019;106:61–8. 30471649 10.1016/j.ejca.2018.10.002

[7] Robinson B Schlumberger M Wirth LJ. Characterization of tumor size changes over time from the phase 3 study of lenvatinib in thyroid cancer. J Clin Endocrinol Metab 2016;101:4103–9. 27548104 10.1210/jc.2015-3989 PMC5095235

[8] Schlumberger M Tahara M Wirth LJ. Lenvatinib versus placebo in radioiodine-refractory thyroid cancer. N Engl J Med 2015;372:621–30. 25671254 10.1056/NEJMoa1406470

